# A pregnant woman with a giant bilateral parietal hemangiopericytoma underwent multiple surgeries and radiotherapy: a case report and literature review

**DOI:** 10.3389/fonc.2023.1172143

**Published:** 2023-07-24

**Authors:** Yingxi Wu, Yafei Xue, Xinqi Wang, Junting Li, Yan Qu, Tianzhi Zhao

**Affiliations:** ^1^ Department of Neurosurgery, Tangdu Hospital, Air Force Medical University, Xi’an, Shaanxi, China; ^2^ Department of Pathology, Tangdu Hospital, Air Force Medical University, Xi’an, Shaanxi, China

**Keywords:** intracranial hemangiopericytoma, pregnant woman, surgical resection, gamma knife radiosurgery, histopathology, malignant transformation

## Abstract

Intracranial hemangiopericytoma is a rare invasive tumor originating from mesenchymal fibroblasts and is prone to local recurrence and distant metastasis. This study reports a case of a 27-year-old woman who presented with severe headache, nausea and vomiting for two weeks at thirty-three weeks of gestation. Cranial magnetic resonance imaging (MRI) demonstrated a giant lesion in the bilateral parietal lobe with a size of 5.12x9.19x6.03 cm and severe edema in the surrounding brain tissue. The patient underwent four operations and 3 gamma knife radiosurgery procedures and is recovering well now. The histopathology findings showed hemangiopericytoma and STAT6 and CD34 positivity after the first and second surgeries. Because of tumor progression, the patient received gamma knife radiosurgery at 1, 3, and 4 years after the first operation. Total tumor resection was achieved in the fourth surgery. Nevertheless, the patient showed malignant transformation to from low-grade to high-grade hemangiopericytoma.

## Introduction

It is infrequent for women to have central nervous system tumors during pregnancy. Many studies have stated that sexual hormones may influence the development of tumors (1–3), but some other studies have indicated that sexual hormones are not associated with the development of intracranial lesions (4). Physiological changes during pregnancy can worsen intracranial hypertension and other clinical symptoms caused by intracranial lesions (5, 6). In addition, neurosurgical disorders place pregnant women and fetuses at risk and complicate treatment.

Hemangiopericytomas are very rare intracranial vascularized mesenchymal neoplasms that are aggressive and have a high propensity for local recurrence and distant metastasis (7–9). Because the stress of anesthesia and surgery may result in fetal or maternal compromise, special care should be taken when performing surgery on pregnant women. Additionally, due to the rich blood supply to tumors and invasion to adjacent tissue, it is highly challenging for neurosurgeons to resect hemangiopericytomas. Furthermore, there was no treatment consensus for pregnant women with intracranial tumors. We introduced a pregnant woman with a giant hemangiopericytoma and summarized our surgical strategy and treatment experience.

## Case presentation

**Figure 1:**
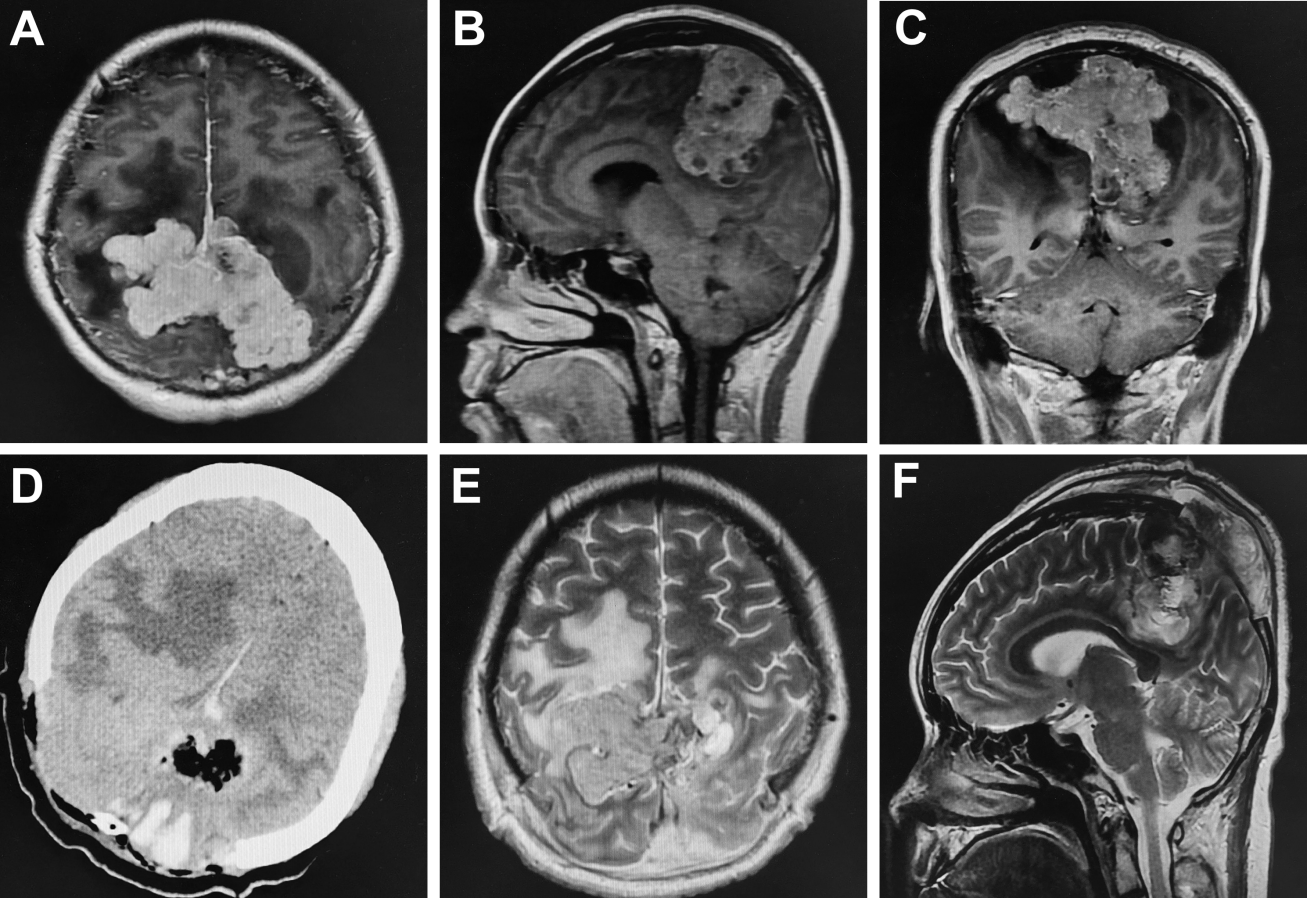
Comparison of preoperative and postoperative imaging. Preoperative enhanced MRI showed heterogeneous enhancement in the bilateral frontal-parietal with a size of 5.12x9.19x6.03cm; the adjacent brain tissue and ventricle were significantly compressed and there was obvious peritumoral edema **(A–C)**. Postoperative cranial CT and MRI demonstrated tumor was partially resected **(D–F)**.

A 27-year-old pregnant woman had an intermittent headache for three months, which was aggravated for two weeks and accompanied by nausea and vomiting. Brain MRI showed equal T1 and long T2 signal shadows in the bilateral frontal-parietal lobe; after enhancement, an uneven mass with a size of 5.12x9.19x6.03 cm was seen (**Figures 1A–C**). The adjacent brain tissue and ventricle were significantly compressed, and there was obvious peritumoral edema (**Figures 1A–C**). Due to the headache, vomiting and other symptoms of high intracranial pressure, the gestational period of the fetus could not be prolonged. Therefore, on the second day after admission, a cesarean section was performed under continuous epidural anesthesia. After the cesarean section, the fetus survived, and the woman was given oxytocin to promote uterine contraction. Because the woman still had severe nausea and vomiting, bilateral parietal lesions were removed under general anesthesia on the 4th day after the cesarean section after a discussion between the neurosurgeons. During the operation, it was found that the tumor had seriously eroded the skull, it was closely adhered to the dura mater, and it had an unclear boundary with the surrounding brain tissue. Furthermore, the tumor had an extremely rich blood supply, leading to excessive intraoperative bleeding, so partial resection was performed. A relaxation suture was applied to the dura mater, and a bone flap was removed. The intraoperative blood loss was 6500 ml, and seventeen units of red blood cells, 2400 ml plasma and two units of cryoprecipitate were infused into the woman. The clinical symptoms were relieved after the operation. Postoperative imaging showed the tumor was partially removed (**Figures 1D–F**). The histopathology indicated hemangiopericytoma (WHO grade I), and immunohistochemistry staining showed that the tumor cells were positive for STAT6 and CD34.

**Figure 2:**
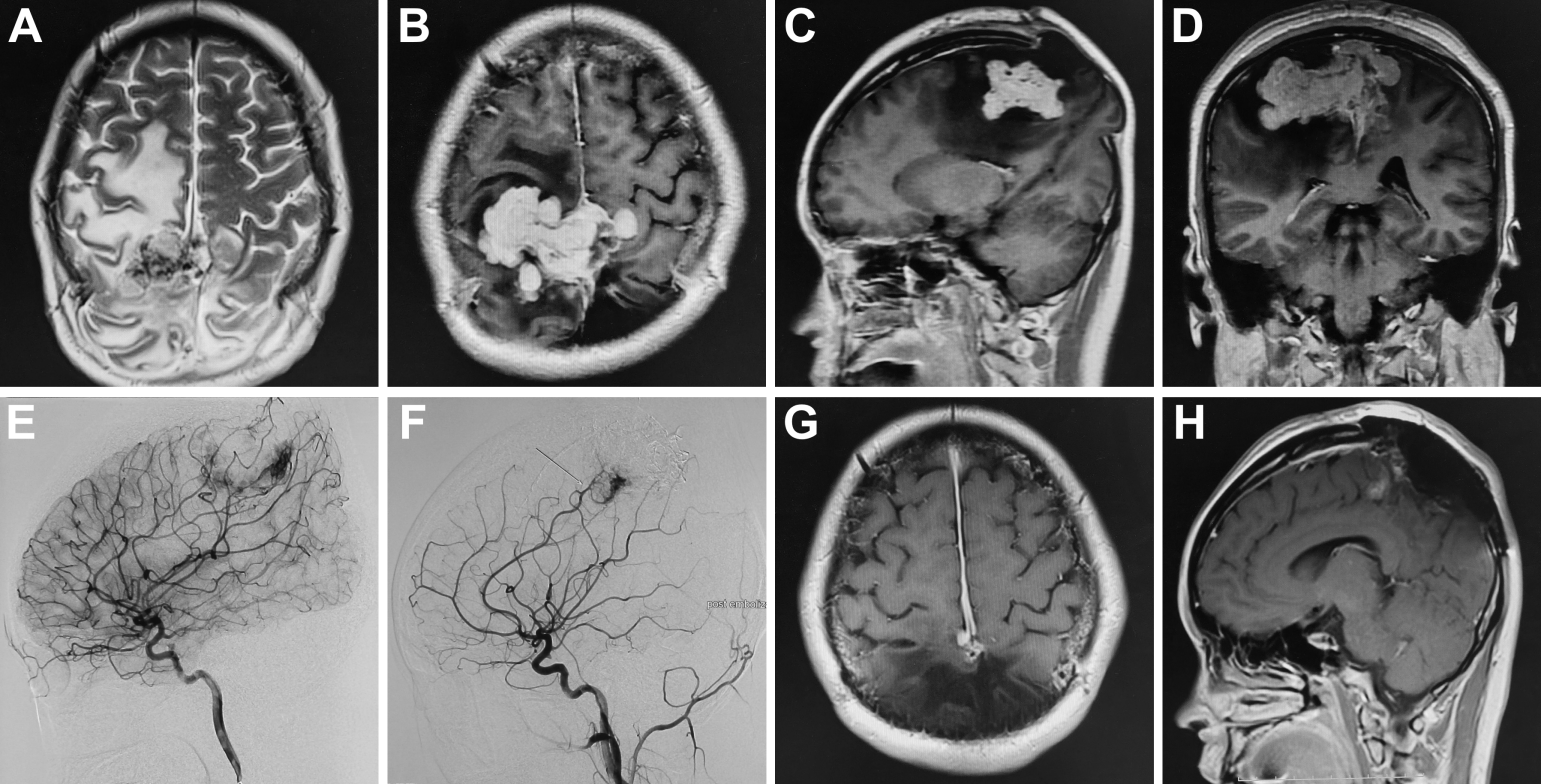
Preoperative MRI showed slight long T2 signal shadow in the frontal-parietal lobe; after enhancement, the lesion demonstrated heterogeneous enhancement with a size of 7.1x5.0x3.7**(A–D)**. Preoperative cerebral angiography indicated that the bilateral middle meningeal artery mainly supplied the tumor **(E)**; after the embolization, cerebral angiography showed that the bilateral middle meningeal artery no longer supplied blood to the tumor, and the tumor staining became weak **(F)**. Postoperative MRI indicated the tumor was near-totally resected **(G, H)**.

Two months after the first operation, the patient was hospitalized again with an intermittent headache. Preoperative MRI showed equal T1 and slight long T2 signal shadow in the frontal-parietal lobe; after enhancement, the lesion demonstrated heterogeneous enhancement with a size of 7.1x5.0x3.7 cm (**Figures 2A–D**). Cerebral angiography showed that the bilateral middle meningeal artery mainly supplied the tumor, and the right middle cerebral artery branch, bilateral posterior cerebral artery branch and left anterior cerebral artery branch participated in the blood supply (**Figure 2E**). First, embolization of the bilateral middle meningeal artery was performed under general anesthesia, and Onyx glue was injected into the bilateral middle meningeal artery through a microcatheter. Cerebral angiography showed that the bilateral middle meningeal artery no longer supplied blood to the tumor, and the tumor staining became weak (**Figure 2F**). Then, the operation was performed along the original incision, and the scalp, subcutaneous tissue and muscle layers were cut layer by layer. After electrocoagulation of the bleeding point on the dura mater that was cut in a “+” shape, the frontal-parietal brain tissue bulged outward. Under the microscope, the tumor was grayish-yellow, tough, and tightly adhered to the brain tissue. First, part of the tumor in the tumor cavity was removed, and then the tumor tissue along the interface between the tumor and brain tissue was separated. Finally, postoperative imaging showed that the tumor was nearly completely removed (**Figures 2G, H**). The woman had a good postoperative recovery and underwent cranioplasty five months after the first operation.

**Figure 3:**
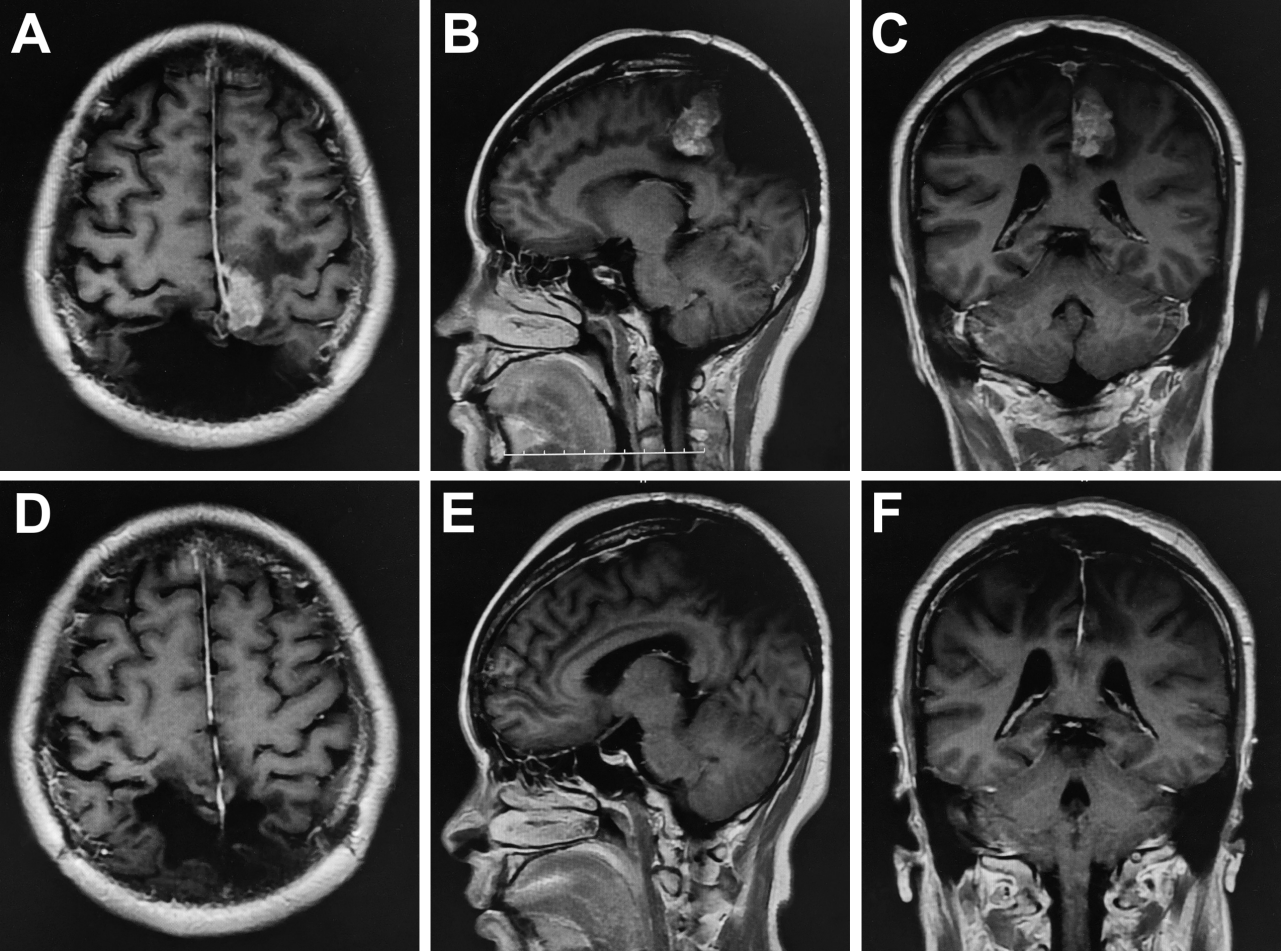
Preoperative enhanced MRI showed that the nodular enhancement was found in both sides of parietal cerebral falx, with the largest size of 3.4x1.8x3.6 cm **(A–C)**. Postoperative MRI demonstrated the tumor was resected completely **(D–F)**.

**Figure 4:**
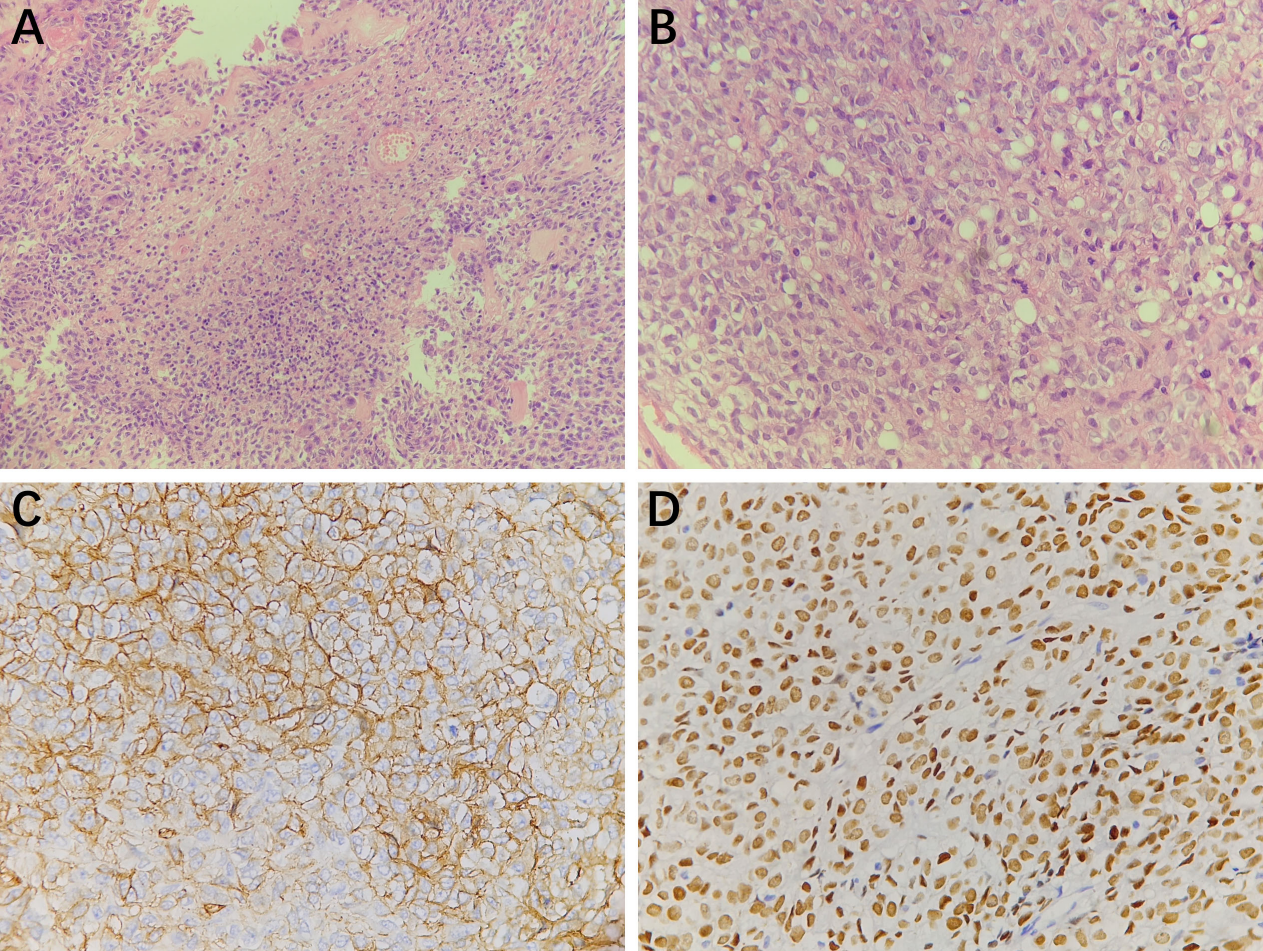
Histopathology results (**A**, 200×magnification and **B**, 400×magnification) showed the tumor cells were round and oval with visible nucleoli, mitotic figures were easy to be seen, and small focal necrosis could be seen. Thin-walled blood vessels could be observed in the stroma. Immunohistochemistry staining at 400× magnification showed that the tumor cells were positive for CD34 **(C)**, STAT6 **(D)**.

One year after the first operation, cranial MRI showed that the tumor had progressed, and the woman was treated with gamma knife radiosurgery. Treatment planning was made by MRI imaging, and two target regions and two targets were designed. We used 14Gy at the 40% isodose line with a maximal center dose of 35 Gy. In the third and fourth years after the first operation, the patient received gamma knife treatment again due to tumor progression. We designed one target region and three targets and used 12Gy at the 40% isodose line with a maximal center dose of 30 Gy. In the fifth year after the first operation, the tumor recurred again, and the MRI demonstrated that part of the parietal brain tissue was lost and patchy irregular long T1 signal shadow was seen in the bilateral parietal lobe. After enhancement, the nodular enhancement was found in both sides of parietal cerebral falx, with the largest size of 3.4x1.8x3.6 cm (**Figures 3A–C**). The tumor was completely resected together with a thickened cerebral falx around the tumor (**Figures 3D–F**). After tumor resection, the dura mater was carefully sutured, the titanium plate was returned, and an epidural drain was placed. Histopathology examination suggested anaplastic hemangiopericytoma (WHO grade III, **Figures 4A, B**), immunohistochemistry staining also showed that the tumor cells were positive for STAT6 and CD34 (**Figures 4C, D**).

## Discussion

Intracranial tumors reported in gravid patients are relatively rare and include gliomas, meningioma, cerebellar hemangioblastoma, and acoustic neuromas (6, 10, 11). Owing to the rarity of hemangiopericytomas, this report describes a rare case of a woman who had hemangiopericytomas during pregnancy. To date, only three cases have been reported in the literature (12–14). Surgeons need to consider aggressive surgical treatment for pregnant women based on the severity of the patient’s symptoms, the pathological nature of the tumors, and the safety of the fetus.

Hernández-Durán et al. (12) reported a 23-year-old pregnant woman with intracranial hypertension who underwent partial tumor resection by a right suboccipital approach at 22 weeks of gestation and underwent a cesarean section in the 36th week. At the last follow-up, the mother and the baby were in good health. Annunziato et al. (13) reported a 38-year-old pregnant woman complaining of severe diplopia for two weeks who underwent total tumor resection by a fronto-temporal approach after delivery. During the second pregnancy, the tumor recurred, the patient underwent reoperation, and complete resection was obtained after the second delivery. Ju et al. (14) also described a 26-year-old pregnant woman at 34 + 4 weeks with severe nausea and vomiting who underwent a cesarean section and tumor removal under general anesthesia at the same time and recovered well. After the operation, the patient received fractionated radiotherapy, and no recurrence or metastasis was found during the six-month follow-up.

We reported a pregnant woman with headache, nausea and vomiting for two months, and she did not pay attention to these symptoms. Most women have nausea and vomiting in the first trimester of pregnancy that are easily neglected, but when severe headache and vomiting with electrolyte imbalance are encountered, intracranial disease needs to be considered. The patient was treated with strong dehydration drugs and was unable to eat food due to increased intracranial pressure. Considering that the patient was at 33 weeks of gestation and had a large intracranial tumor and after discussion between neurosurgeons and obstetricians, it was decided to perform cesarean section first and then perform intracranial lesion resection. After the cesarean section, the woman still had an obvious headache, so surgery was performed under general anesthesia on the 4th day after the cesarean section. Among the three cases described above, one patient underwent craniotomy before cesarean section, one underwent craniotomy after delivery, and one underwent cesarean section and craniotomy simultaneously.

Because the case of hemangiopericytoma was a highly vascularized tumor, the intraoperative blood loss was more than 6500 ml, and the surrounding brain tissue had prominent edema. Therefore, subtotal resection was performed, and the bone flap was removed. The patient’s symptoms were significantly relieved after the operation. It has been reported that vascular embolization can reduce intraoperative bleeding and increase the degree of tumor resection (15, 16). Given the rich blood supply of this tumor, bilateral middle meningeal artery embolization was performed before the second operation to reduce intraoperative bleeding, and near total resection was obtained.

Many studies have indicated that surgery combined with adjuvant radiotherapy can prolong progression-free survival but not overall survival (8, 17–20). The woman in this case developed tumor progression at 1, 3, and 4 years after the first operation, and she received gamma knife radiosurgery after each tumor progression. Because the gamma knife radiosurgery can more accurately locate the residual small lesions, giving a high radiation dose at one time, and could minimize the damage to surrounding brain tissue. In addition, repeat gamma knife treatment can still be used to treat recurrent hemangiopericytoma (21). In the three patients mentioned above, only one patient experienced postoperative radiotherapy, and the other two patients did not experience postoperative radiotherapy. All of the four patients did not receive any chemotherapy medication.

In our patient, after the fourth tumor progression, the woman underwent a third tumor resection and total tumor resection was obtained. However, the pathological results showed high-grade hemangiopericytoma (WHO grade III) and STAT6 and CD34 positivity, and STAT6 positivity is a specific immunohistochemical marker of hemangiopericytoma (22). Nevertheless, the specimens from the first and second surgical resection revealed low-grade hemangiopericytoma (WHO grade I), which indicated the tumor developed malignant progression from low-grade to high-grade based on the 2021 WHO classification of central nervous system tumors. Moreover, the high-grade hemangiopericytoma showed a mitotic count of more than 5 mitoses/10 HPF with necrosis, and the Ki-67 index increased to 20%. Apra et al. reported that in 18 recurrent solitary fibrous tumors/hemangiopericytomas, 5 patients developed malignant progression from grade I or to grade III (23). Zhao et al. also pointed out that two of five patients with recurrence had pathological malignant transformation in their study (24).

In conclusion, pregnant women with primary hemangiopericytomas are very rare, and diagnosing hemangiopericytomas is also difficult. Given the high risk of resecting giant hypervascular hemangiopericytomas and the safety of the mother and fetus, surgical resection of the tumor must be performed meticulously and carefully. In addition, patients with tumor progression after surgery could be treated with a repeat gamma knife, which can improve progression-free survival. Furthermore, after the third tumor removal, we found that the tumor had developed a malignant transformation from WHO grade I to grade III based on the 2021 WHO classification.

## Data availability statement

The original contributions presented in the study are included in the article/supplementary material. Further inquiries can be directed to the corresponding authors.

## Ethics statement

The studies involving human participants were reviewed and approved by Tangdu Hospital of Air Force Military Medical University. The patients/participants provided their written informed consent to participate in this study. Written informed consent was obtained from the individual(s) for the publication of any potentially identifiable images or data included in this article.

## Author contributions

TZ and YQ: chief surgeon, writing—review and editing. YW and YX: writing—original draft preparation. XW and JL: data collection and analysis. All authors contributed to the article and approved the submitted version.
